# Anticancer effects of 4-vinyl-2,6-dimethoxyphenol (canolol) against SGC-7901 human gastric carcinoma cells

**DOI:** 10.3892/ol.2013.1230

**Published:** 2013-03-05

**Authors:** JING JIANG, DONG-HUI CAO, TETSUYA TSUKAMOTO, GUO-QING WANG, ZHI-FANG JIA, JIAN SUO, XUE-YUAN CAO

**Affiliations:** 1 Division of Clinical Epidemiology, First Hospital of Jilin University, Changchun, Jilin 130021, P.R. China;; 2 Division of Pathology, School of Medicine, Fujita Health University, Toyoake, Japan;; 3 Department of Pathogeny Biology, Norman Bethune Medical College of Jilin University, Changchun, Jilin 130021, P.R. China; 4 Department of Gastric and Colorectal Surgery, First Hospital of Jilin University, Changchun, Jilin 130021, P.R. China

**Keywords:** canolol, gastric cancer, COX-2, anti-proliferation, apoptosis

## Abstract

Gastric cancer remains the fourth most commonly diagnosed cancer and is the second leading cause of cancer-related mortality worldwide. The aim of this study was to investigate the effects of canolol on the proliferation and apoptosis of SGC-7901 human gastric cancer cells and its relevant molecular mechanisms. The 3-(4,5-dimethylthiazol-2-yl)-2,5-diphenyltetrazolium bromide (MTT) assay was used to observe the effect of canolol on the proliferation of SGC-7901 human gastric adenocarcinoma cells. The results showed that SGC-7901 cells exhibited a marked dose-dependent reduction in the proliferation rate. The survival rate of the cells was 88.86±1.58% at 50 *μ*mol/l, decreasing to 53.73±1.51% at 800 *μ*mol/l (P<0.05). By contrast, canolol had no significant toxicity on the human gastric mucosal epithelial cell line GES-1. The vivid images of cell morphology using an inverted microscope provided confirmation of the MTT assay. Treatment of SGC-7901 cells with canolol resulted in apoptosis demonstrated by flow cytometry. Furthermore, canolol downregulated the mRNA levels of COX-2, but had no significant effect on the mRNA expession of the Bax and Bcl-2 genes. These findings suggest that canolol has potential to be developed as a new natural anti-gastric carcinoma agent.

## Introduction

Gastric cancer remains the fourth most commonly diagnosed cancer and is the second leading cause of cancer related mortality worldwide (1). Gastric cancer is the most common cancer in Eastern Asia (2). Eradication of *H. pylori* in the stomach by administration of oral antimicrobial agents results in the resolution of *H. pylori*-infected chronic active gastritis and significantly reduces the risk of gastric cancer development (3). However, bacterial eradication treatment has been lacking. The occurrence of antibiotic-resistant *H. pylori* has been reported (4) and is occasionally associated with adverse effects. Regular therapies such as chemotherapy, biotherapy and radiotherapy have been previously applied, however, they have unavoidable side effects (5). Therefore, more effective alternative approaches for gastric cancer prevention and therapies without undesirable side-effects are needed.

It is widely accepted that phytochemical, especially phenolic, compounds are associated with anticancer effects by affecting molecular events in the initiation, promotion and progression stages. Recent studies have demonstrated protective effects of plant phenolic compounds against gastric cancer (6–8). The expansion ability of tumor cells depends on the rate of both cell proliferation and cell apoptosis. The particular features of tumor cells allow them to evade apoptosis, a cell suicide program that reduce the damaged or mutated cells to maintain homeostasis (9).

**Figure 1:**
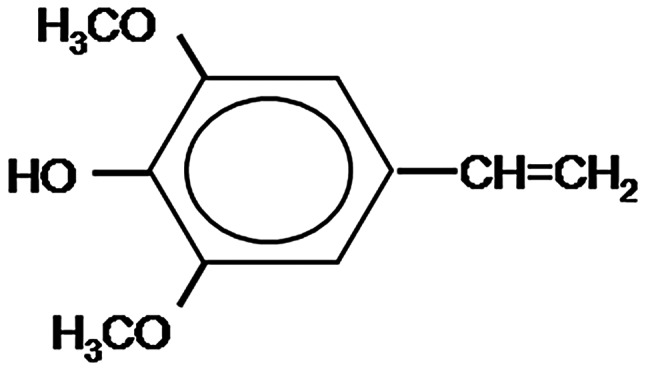
Chemical structure of canolol, 4-vinyl-2,6-dimethoxyphenol. Molecular weight: 180.

Canolol, 4-vinyl-2,6-dimethoxyphenol (Fig. 1), is purified from crude canola oil and is a novel and potent antioxidant. Canolol has been proven to prevent *H. pylori*-induced gastritis and carcinogenesis in an animal model (10). However, its potential anti-proliferative and proapoptotic effects on gastric cancer cells and the possible mechanisms remain unknown.

The role of cyclooxygenase-2 (COX-2) inhibitors in the chemoprophylaxis of gastric cancer has been investigated. COX-2, the inducible isoform of COX, is undetectable in normal tissues and highly expressed in gastric tumors (11). Experimental studies have identified the correlation between COX-2 overexpression and the increased cell proliferation and decreased cell apoptosis in malignant tumor cells (12,13). COX inhibitors (Coxibs) are a series of drugs with analgesic, antipyretic and anti-inflammatory properties. Evidence suggests that COX-2 inhibitors correlate with tumor inhibition in breast (14) and endometrial cancer cell lines (15). Induction of apoptosis has increasingly become important with regard to the mechanism of cancer defense and prevention (16). However, the involvement of COX-2 inhibitors in gastric cancer prophylaxis remains to be determined, as the long-term use of COX-2 inhibitors exerts side-effects on the cardiovascular system and the digestive tract. A possible correlation between COX-2 inhibition and cell apoptosis in gastric cancer cell lines has yet to be examined.

In the present study, the effects of canolol on growth and apoptosis of human gastric adenocarcinoma SGC-7901 cells were investigated. Human gastric mucosal epithelial (GES-1) cells were used as the control cell model to examine the non-specific cytotoxicity of canolol. The mRNA expression levels of COX-2, Bcl-2 and Bax were detected to further elucidate the possible mechanisms involved.

## Materials and methods

### Materials and reagents

4-Vinyl-2,6-dimethoxyphenol (canolol with a molecular mass of 180) was purchased from Junsei Chemical, Tokyo, Japan. It was synthesized to at least 95% purity (confirmed by nuclear magnetic resonance). The preparation was sealed under helium or nitrogen and maintained at −80°C. Canolol was dissolved in ethanol and diluted in a serum-free medium immediately before the experiments. Gastric cancer SGC-7901 cells were obtained from the Department of Pathogeny Biology, Norman Bethune Medical College of Jilin University, China. Human gastric mucosal epithelial cell line GES-1 was obtained from the Cancer Hospital of Beijing University. The study protocol was approved by the ethics committee of the First Hospital of Jilin University.

### Cell culture and treatment

Human SGC-7901 gastric cancer cell line and human GES-1 gastric mucosal epithelial cell line were cultured in RPMI-1640 medium containing 10% heat-inactivated fetal bovine serum (FBS) and 100 ng/ml each of penicillin and streptomycin in an incubator (50 ml/l CO_2_) at 37°C. The medium was changed every 2–3 days. Cells in the logarithmic growth phase were collected for subsequent experiments. The cells were treated with various concentrations of canolol for 24 h.

### Cell viability assay

The method of 3-(4,5-dimethylthiazol-2-yl)-2,5-diphenyltetrazolium bromide (MTT) assay was employed to determine cell viability. Cultured SGC-7901 and GES-1 cells were detached using trypsinization, centrifuged at 1,000 × g for 5 min and resuspended in fresh RPMI-1640 medium. The cells were plated at a density of 5×10^3^ cells/well in 96-well microplates and treated with canolol ranging from 25 to 1,200 *μ*mol/l for 24 h at 37°C. At the end of treatment, 20 *μ*l of MTT stock solution was added to each well [(0.5 mg/ml in phosphate-buffered saline (PBS)] for 4 h. The medium was replaced with 150 *μ*l DMSO to dissolve the converted purple dye in the culture plates. Absorbance was measured at 570 nm on a spectrophotometer microplate reader. Cell viability was assayed as the relative formazan formation in treated compared with control wells after correction for background absorbance. Four wells per dose were counted in each experiment. Analyses were performed using SPSS version 10.0 (SPSS Inc, Chicago, IL, USA). Data were evaluated using one-way ANOVA. P<0.05 was considered statistically significant.

### Cell morphology

SGC-7901 and GES-1 cells were seeded at a density of 5×10^5^ cells/well onto a cover slip loaded in 6-well plates. Fresh RPMI-1640 medium containing different concentrations of canolol was added. Cells were photographed with an inverted microscope under ×200 magnifications to observe morphological changes.

### Annexin V-FITC/PI staining for flow cytometry

SGC-7901 cells were collected and centrifuged at 1,000 × g for 5 min and resuspended in fresh RPMI-1640 medium at a density of 2×10^5^ cells/ml. Apoptotic and necrotic cells were evaluated by Annexin V (AV) binding and propidium iodide (PI) uptake using an AV-FITC-PI apoptosis assay kit (Pharmingen, San Diego, CA, USA). Samples were analyzed by flow cytometry.

### Real-time quantitive PCR analysis

Total RNA of SGC-7901 cells was extracted using an RNA extraction kit and primers used are shown in Table I. Following DNase treatment, the first strand cDNA was synthesized. Quantitive PCR of Bcl-2, Bax and COX-2 were performed with the Bio-Rad (Hercules, CA, USA) CFX system. To exclude variations caused by RNA quantity and quality, the GAPDH gene was used as an internal control. Analyses were performed using SPSS version 10.0 (SPSS Inc). Data were evaluated using one-way ANOVA. P<0.05 was considered a statistically significant result.

## Results

### Canolol does not exhibit evident toxicity to GES-1 cells

**Figure 2:**
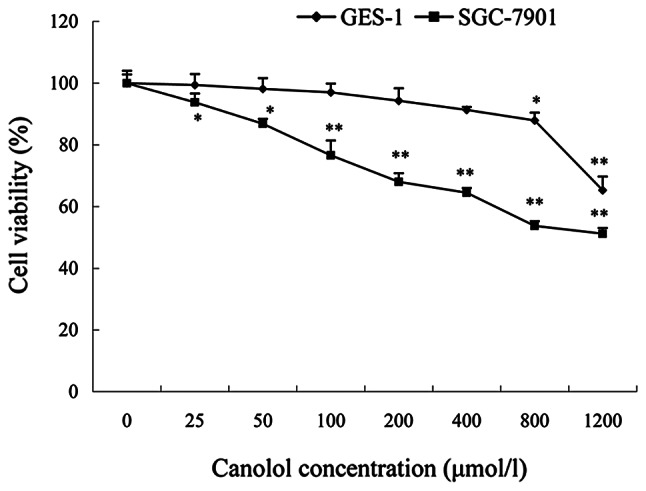
Effect of canolol on cell viability under different concentrations on particular cells using 3-(4,5-dimethylthiazol-2-yl)-2,5-diphenyltetrazolium bromide (MTT) assay (mean ± SD) (n = 4). Data were evaluated using oneway ANOVA. ^*^P<0.05, ^**^P<0.01.

**Figure 3:**
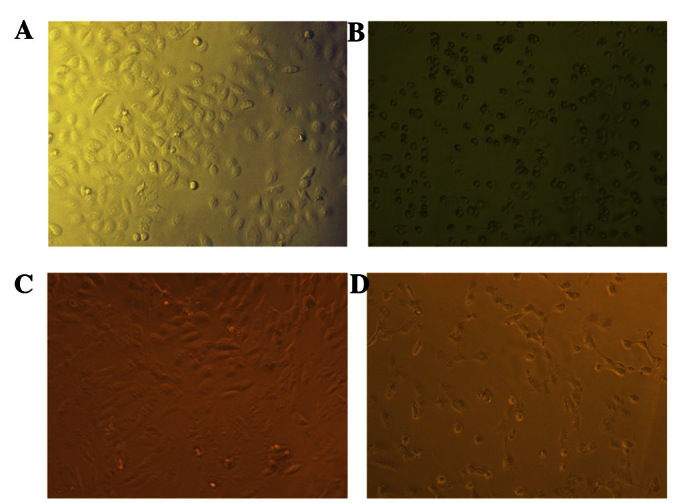
Morphology of GES-1 and SGC-7901 cells treated with 1,200 *μ*mol/l canolol.

The proliferation effect of canolol was determined using an MTT assay and GES-1 cells were used as a control to detect the cell toxicity of canolol. Cells were treated with different concentrations of canolol (0–1200 *μ*mol/l). The data indicated that canolol has no obvious cytotoxicity against normal GES-1 cells. The percentage of cell viability was 99.38±3.57% at 25 *μ*mol/l, 87.82±2.55% at 800 *μ*mol/l and decreased to 65.31±4.44% at 1200 *μ*mol/l (Fig. 2). Cell morphology using an inverted microscope also showed that cell structures were intact and were well established after 1,200 *μ*mol/l canolol treatment (Fig. 3).

### Canolol inhibits proliferation and induces apoptosis of SGC-7901 cells

SGC-7901 cells were treated with different concentrations of canolol (0–1200 *μ*mol/l). The percentages of cell viability at various canolol doses were determined as the percentage of viable treated cells in comparison with viable untreated cells. The results provided solid evidence that the inhibitory effects on the proliferation of canolol to SGC-7901 cells were dose-dependent (Fig. 2); the percentage of cell viability was 89.80±2.83% at 25 *μ*mol/l, 73.73±1.51% at 800 *μ*mol/l (P*<*0.05) and 51.22±1.82% at 1,200 *μ*mol/l (P*<*0.01). Consistent with the MTT assay results, the adherent SGC-7901 cells were markedly decreased and showed apoptosis under the treatment of 1,200 *μ*mol/l canolol (Fig. 3).

**Figure 4:**
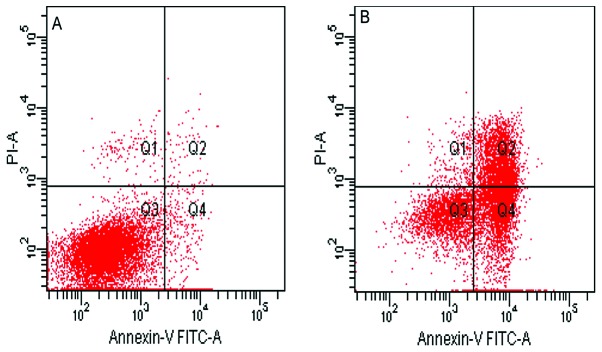
Apoptosis of SGC-7901 cells was investigated using a flow cytometry assay using FITC-Annexin-V/PI staining. (A) SGC-7901 cells without canolol; (B) SGC-7901 cells with 400 *μ*mol/l canolol.

Furthermore, a flow cytometric analysis was used to quantify the rate of cell apoptosis using double staining of Annexin V-FITC and PI. As shown in Fig. 4, the lower right field (high Annexin V, low PI staining) represents the early apoptotic cells due to the strong affinity of Annexin V-FITC with phosphatidylserine, which transports from the inner to the outer surface of the plasma membrane during early apoptosis. By contrast, the higher left field (high PI, low Annexin V staining) represents the necrotic cells, since PI, which binds to nucleic acids, only cross through the compromised membrane of dead cells or late apoptotic cells (17). Viable cells are shown in the lower left field (low Annexin V and PI staining) and the higher right field (high Annexin V and PI staining), indicating late apoptotic cells. The results showed that canolol was able to induce the apoptosis of SGC-7901 cells and the rate of early apoptosis, late apoptosis and necrosis of SGC-7901 cells were increased under 400 *μ*mol/l canolol (Fig. 4).

### Canolol downregulates the mRNA expression level of COX-2

**Figure 5:**
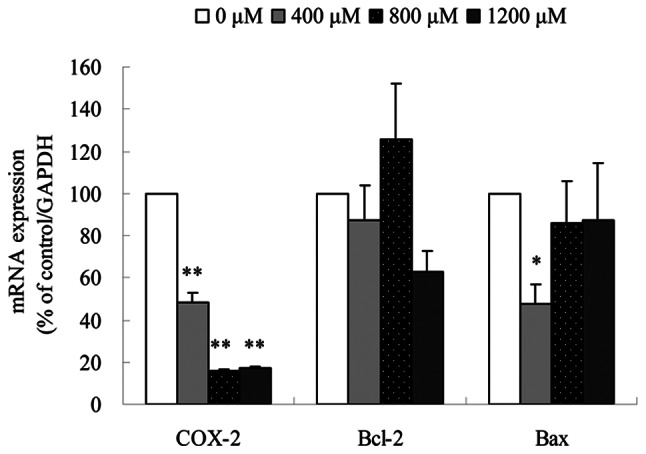
Relative expression levels of COX2, Bcl-2 and Bax mRNAs in SGC-7901 cells under treatment of different concentrations of canolol (mean ± SD) (n=3). Values are arbitrary unit values (mean ± SD) relative to 100 for controls. GAPDH was used as an internal control. Data were evaluated using one-way ANOVA. ^*^P<0.05, ^**^P<0.01 vs. control.

To clarify the mechanisms of SGC-7901 cell apoptosis under canolol treatment, the mRNA expression level of COX-2, Bcl-2 and Bax was evaluated using real-time quantitive PCR. The sequences of these primers were shown in Table I. The results showed that in SGC-7901 cells, the relative mRNA expression level of COX-2 was decreased to 48.50±4.67% in 400 *μ*mol/l, 16.08±0.75% in 800 *μ*mol/l and 17.22±0.88% in 1,200 *μ*mol/l canolol. The effect of canolol on COX-2 expression was downregulated (P*<*0.01); However, the expression levels of Bcl-2 and Bax fluctuated slightly (Fig. 5). These data suggested that the inhibition of COX-2 might play an important role in the apoptosis of SGC-7901 cells.

## Discussion

Gastric cancer is one of the most prevalent malignant tumors and its morbidity is the highest in China. Currently, many natural and synthesized compounds are used in the chemoprovention and treatment of gastric cancer (18,19). Canolol, 4-vinyl-2,6-dimethoxyphenol, which is extracted from crude canola oil, has the ability to prevent *H. pylori*-infected gastric carcinogenesis in gerbils (10). In the present study, it was demonstrated that canolol prevented proliferation and induced apoptosis of SGC-7901 cells dose-dependently *in vitro*. Additionally, it had low toxicity to immortalized GES-1 cells (Figs. 2 and 3). The results indicated that canolol has the potential to be developed as a new natural anti-gastric carcinoma agent.

COX-2 is important in the conversion of arachidonic acid to prostaglandin H_2_. Accumulating evidence suggests that the constitutive overexpresion of the inducible COX-2 gene is involved in a diverse array of cancers and Harris *et al*(20) demonstrated that COX-2 overexpresion initiated and promoted carcinogenesis through: i) mutagenesis, i.e., the production of certain reactive oxygen species that are carcinogenic; ii) mitogenesis, i.e., cell proliferation promoted by PGE-2 and other factors; iii) anti-apoptosis, i.e., cell differentiation and apoptosis reduced by PGE-2 and other factors; iv) angiogenesis, metastasis and immunosuppression (20). The real-time quantitive PCR in this study showed that COX-2 expression was downregulated under canolol treatment (P*<*0.01) (Fig. 5). It was postulated that inhibition of COX-2 expression may result in blockade of the prostaglandin cascade and a decrease in reactive oxygen species (ROS), thus stimulating apoptosis of malignant cells and preventing neoplastic growth. The scavenging potency of canolol against ROO^•^ is much higher than that of well-known antioxidants, such as α-tocopherol, vitamin C and β-carotene (21). A previous study in this laboratory showed canolol decreased serum 8-OHdG, a key biomarker of oxidative DNA damage relevant to carcinogenesis (10). Other natural phenolic extracts, such as BCE (black currant extract) and dioscin, reduce the risk of gastric cancer owing to their antioxidative functions (22–24).

Selective and non-selective COX-2 inhibitors may be involved in the intervention and chemoprevention of carcinogenesis (25–27). A series of epidemiologic studies found that the COX-2 inhibition levels of coxibs were consistent with their chemopreventive effects in cancers of the breast, colon, prostate and lung (20). Ma *et al*(28) have demonstrated that PGE2 acts with a family of G-protein-coupled receptors participating in multiple signal tranduction pathways.

The Bcl-2 family, such as Bax, Bad, Bid, Bcl-2 and Bcl-x, is one of the most extensively studied groups of proteins involved in cell apoptosis. Bax, Bad and Bid were shown to activate apoptosis, while Bcl-2 and Bcl-x were shown to inhibit the process (29). Transfection of COX-2 constitutive expression vector into the BCC cell line significantly upregulated Bcl-2 expression and this indicated that Bcl-2 might participate in COX-2 mediated anti-apoptic processes (30). In addition, the expression level of Bax, a member of a pro-apopotic protein family was downregulated in a transgenic mouse model (31). However, in the present study, no correlation between Bcl-2/Bax and COX-2 expression was found (Fig. 5).

The relationship between apoptosis and COX-2 downregulation in this gastric adenocarcinoma cells should be studied. COX-2 is a potential pharmacologic target that may be used in the prevention and treatment of various types of malignancies.

## Figures and Tables

**Table I:** Primer sequences used in real-time quantitive PCR.

Gene	Primer sequence	Annealing temperature (°C)	Product size (bp)
COX-2	F: CTCCCTTGGGTGTCAAAGGTA	76	171
	R: GCCCTCGCTTATGATCTGTC		
Bcl-2	F: GAGTTCGGTGGGGTCATG	83	186
	R: GGAGAAATCAAACAGAGGC		
Bax	F: GGATGCGTCCACCAAGAA	83.5	388
	R: GAGCACTCCCGCCACAAA		
GAPDH	F: AACGGATTTGGTCGTATTG	78.5	258
	R: GGAAGATGGTGATGGGATT		

GAPDH, glyceraldehyde-3-phosphate dehydrogenase; F, forward; R, reverse.
